# Associations of Wnt5a expression with liver injury in chronic hepatitis B virus infection

**DOI:** 10.1186/s12879-023-08865-x

**Published:** 2023-12-07

**Authors:** Xiang-Fen Ji, Qi Zhou, Jing-Wei Wang, Fei Sun, Shuai Gao, Kai Wang

**Affiliations:** 1 Department of Hepatology, Qilu Hospital (Qingdao), Shandong University, Qingdao, 266035 China; 2 Department of Pediatric Surgery, Qilu Hospital (Qingdao), Shandong University, Qingdao, 266035 China; 3 Department of Hepatology, Qilu Hospital, Shandong University, Jinan, 250012 China; 4 Hepatology Institute of Shandong University, Jinan, 250012 China

**Keywords:** Wnt5a, Liver injury, Chronic Hepatitis B, Chronic Hepatitis B virus Infection

## Abstract

**Background:**

Aberrant Wnt5a expression contributes to immunity, inflammation and tissue damage. However, it remains unknown whether Wnt5a is associated with liver injury in chronic hepatitis B virus (HBV) infection. We aimed to explore the potential role of Wnt5a expression in liver injury caused by chronic HBV infection.

**Methods:**

Wnt5a mRNA levels in peripheral blood mononuclear cells (PBMCs) were analyzed in 31 acute-on-chronic hepatitis B liver failure (ACHBLF) patients, 82 chronic hepatitis B (CHB) patients, and 20 healthy controls using quantitative real-time polymerase chain reaction. Intrahepatic Wnt5a protein expression from 32 chronic HBV infection patients and 6 normal controls was evaluated by immunohistochemical staining.

**Results:**

Wnt5a mRNA expression was increased in CHB patients and ACHBLF patients compared to healthy controls and correlated positively with liver injury markers. Additionally, there was a significant correlation between Wnt5a mRNA expression and HBV DNA load in all patients and CHB patients but not in ACHBLF patients. Furthermore, intrahepatic Wnt5a protein expression was elevated in chronic HBV infection patients compared to that in normal controls. Moreover, chronic HBV infection patients with higher hepatic inflammatory grades had increased intrahepatic Wnt5a protein expression compared with lower hepatic inflammatory grades. In addition, the cut-off value of 12.59 for Wnt5a mRNA level was a strong indicator in predicting ACHBLF in CHB patients.

**Conclusions:**

We found that Wnt5a expression was associated with liver injury in chronic HBV infection patients. Wnt5a might be involved in exacerbation of chronic HBV infection.

## Background

Hepatitis B virus (HBV) infection is a serious public health problem, with approximately 240 million individuals worldwide being chronically infected [1]. Chronic HBV infection can cause immune-mediated inflammation and liver injury, leading to chronic hepatitis B (CHB), and even progression to acute-on chronic liver failure (ACLF) [2]. CHB can be treated with antiviral therapy, but ACLF has a significantly high mortality with few effective treatments [2, 3]. Therefore, monitoring and assessing the severity of liver injury and progression of chronic HBV infection is important in clinical practice. Unfortunately, despite a large number of studies undertaken to further understand the immune response and HBV-associated liver injury [4–6], the mechanism remains obscure.

Wnt proteins are a large family of glycoprotein ligands that play important roles in many cellular functions, including proliferation, differentiation, migration, polarization, and apoptosis [7]. Wnt proteins can be grouped into two distinct classes depending on their downstream involvement or absence of β-catenin: canonical and noncanonical [7, 8]. Noncanonical Wnt5a pathways are usually initiated by binding to frizzled (Fzd) receptors, receptor tyrosine kinase-like orphan receptor (Ror) and receptor tyrosine kinase related tyrosine kinase (Ryk), whereas intracellular pathways are mediated by some molecules, such as Ca2+, c-Jun N-terminal kinase (JNK), calmodulin dependent protein kinase II (CaMKII) and protein kinase C (PKC) [7, 9]. Basal expression of Wnt5a has been observed in many immune cells [10, 11]. Wnt5a, which is dependent on Toll-like receptors, can induce phenotypic changes in monocytes, macrophages and dendritic cells, mediating the innate immune response [12–14]. Additionally, Wnt5a signaling contributes to CD4 T-cell homeostasis and activation of CD8 T cells, which triggers adaptive immunity [15, 16]. Increasing evidence suggests that Wnt5a signaling may play a crucial role in inflammatory diseases [17, 18]. In highly active antiretroviral therapy (HAART)-associated NeuroAIDS, administration of nucleoside reverse transcriptase inhibitors induced inflammatory cytokine production, including interleukin (IL)-6, IL-1β and TNF (tumor necrosis factor)-α, in various regions of the central nervous system via a Wnt5a signaling-dependent mechanism [19]. Wnt5a signaling mediates the pathogenesis of lung inflammation and airway epithelial injury in benzo(a)pyrene-induced pulmonary dysfunction [20]. In addition, Wnt5a is reported to be increased in experimental and human chronic obstructive pulmonary disease (COPD) and to induce production of inflammatory cytokines such as IL-6 and TNF-α [21]. Box5, a Wnt5a antagonist, is a hexapeptide that antagonizes Wnt5a-mediated signaling via direct frizzled class receptor 5 (FZD5) binding [22]. Many Wnt5a-mediated cellular activities, including migration, invasion, and apoptosis can be antagonized by Box5 [22, 23]. Inhibiting Wnt5a signaling with Box5 decreases the expression of C-C motif chemokine 2 (CCL-2), IL-6 and TNF-α, reduces the percentage of F4/80 + macrophage infiltration in the kidney, and alleviates renal injury in diabetic mice [24]. Furthermore, fine particulate matter (PM2.5) induces upregulation of Wnt5a, which promotes human bronchial epithelial cell proliferation, and production of IL-1β, IL-6 and IL-8, which was demonstrated to be prevented by Box5 [25, 26]. Moreover, limiting Wnt5a activity via Box5 reduces stroke size in rats by middle cerebral artery occlusion, eliciting neurological protection [27].

We have previously shown that serum Wnt5a can serve as a predictor for 3-month mortality in liver failure [28]. Furthermore, we demonstrated that Wnt5a expression was enhanced in a mouse model of acute liver failure [29]. Inhibiting Wnt5a signaling with Box5 reduces liver inflammation and injury [29]. In addition, HBV replication was shown to upregulate Wnt5a expression [30]. However, the potential role of Wnt5a as a predictor of liver injury caused by chronic HBV infection remains unknown. In this study, we initially determined mRNA expression of Wnt5a, IL-6, TNF-α and IL-1β in peripheral blood mononuclear cells (PBMCs) from acute-on-chronic hepatitis B liver failure (ACHBLF) patients and CHB patients. Next, we assessed the location and expression of Wnt5a protein in liver tissue. Additionally, we investigated the potential association between Wnt5a expression and the severity of liver injury.

## Methods

### Study design and subjects

**Fig. 1:**
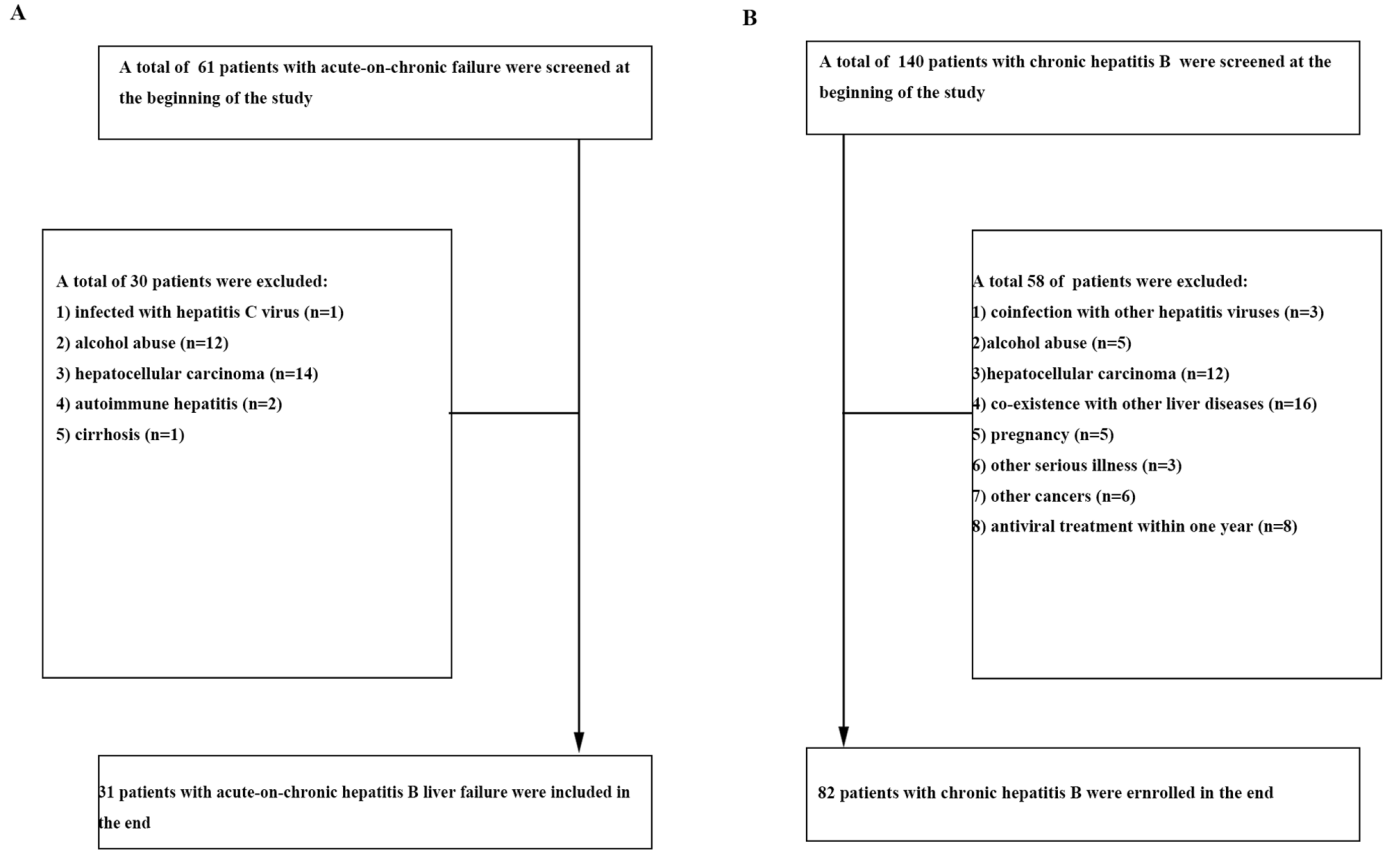
Inclusive procession of ACHBLF patients and CHB patients in this present study was shown in the flowchart

A total of 201 patients, including 140 CHB patients and 61 ACHBLF patients, were retrospectively recruited at the Department of Hepatology, Qilu Hospital (Qingdao) of Shandong University from June 2019 to August 2021. According to inclusion and exclusion criteria, only 82 CHB patients and 31 ACHBLF patients were enrolled in the study. The selection and exclusion of ACHBLF patients and CHB patients is described in Fig. 1. Twenty healthy volunteers were enrolled as healthy controls (HCs). Neither the CHB patients nor the ACHBLF patients displayed evidence of liver cirrhosis. In addition, liver biopsies were obtained from 32 patients with chronic HBV infection, and normal liver tissues were collected from 6 patients with haemangioma who underwent partial hepatectomy and termed normal controls. A pathologist assessed the liver inflammatory grade using the modified histological activity index (HAI). Each subject signed informed consent and was approved by the Ethics Committee in Qilu Hospital (Qingdao) of Shandong University prior to the study.

CHB patients had positive serum hepatitis B surface antigen (HBsAg) for at least six months and exhibited elevation of serum alanine aminotransferase (ALT) (> 40 U/L) [31]. ACHBLF was defined following the guidelines of the Asian Pacific Association for The Study of the Liver (APASL) [32]. Patients meeting any of the following exclusion criteria were excluded: (1) coinfection with hepatitis virus A, C, D, E or G and/or human immunodeficiency virus (HIV); (2) drug hepatitis, autoimmune disease, alcoholic hepatitis, and other liver diseases; (3) any type of cancer and other serious medical illness; (4) receiving any immunotherapy or antiviral treatment at least one year prior to blood collection; and (5) pregnancy.

### Peripheral blood mononuclear cell isolation

PBMCs were obtained from ethylenediaminetetraacetic (EDTA)-anticoagulated venous peripheral blood by Ficoll-Paque Plus (GE Healthcare, Uppsala, Sweden) density gradient centrifugation. The interface mononuclear cells were obtained and washed three times with phosphate-buffered saline.

### RNA extraction and quantitative real-time polymerase chain reaction (PCR)

TRIzol reagent (Sparkjade, China) was used to extract total RNA from PBMCs, and the RNA was reverse transcribed into cDNA using RevertAid First Strand cDNA Synthesis Kits (Fermentas, Vilnius, Lithuania). Real-time PCR was conducted using Blaze Taq™ SYBR® Green qPCR Mix 2.0 (GeneCopoeia, Rockville, MD, USA). The primers used are shown in Table 1. GAPDH served as the internal control. All PCR products were determined using the 2^−ΔΔCt^ method.

**Table 1:** Primers for real-time PCR

Primers	Sequences
Wnt5a	5ʹ-ACCACATGCAGTACATCGGAG-3ʹ
	5ʹ-GAGGTGTTATCCACAGTGCTG-3ʹ
TNF-α	5ʹ-AGCTGCGCAGAATGAGATGAGTT-3ʹ
	5ʹ-CAGATAGATGGGCTCATACC-3ʹ
IL-6	5ʹ-ACCCCTGACCCAACCACAAAT-3ʹ
	5ʹ-AGCTGCGCAGAATGAGATGAGTT-3ʹ
IL-1β	5ʹ-AAACAGATGAAGTGCTCCTTCCAGG-3ʹ
	5ʹ-TGGAGAACACCACTTGTTGCTCCA-3ʹ
GAPDH	5ʹ-GCACCGTCAAGGCTGAGAAC-3ʹ
	5ʹ-TGGTGAAGACGCCAGTGGA-3ʹ

### Immunohistochemical staining

Liver tissues were fixed, embedded, and sectioned at 4 μm thickness. The sections were deparaffinized with a series of progressive xylenes and ethanol baths. Heat-induced antigen retrieval was performed in citrate buffer, followed by endogenous peroxidase activity blocking with 0.3% H_2_O_2_. The sections were incubated with primary antibody against Wnt5a (1:200; Bioss, China) overnight at 4 °C and then with horseradish peroxidase (HRP)-labeled secondary antibody. The Wnt5a protein was stained with diaminobenzideine (DAB). All sections were observed under a light microscope. Immunohistochemical positivity was measured using ImageJ 6.0 software.

### Laboratory indices

The laboratory indices of each subject, including serum levels of ALT, aspartate aminotransferase (AST), total bilirubin (TBIL), albumin (ALB), hepatitis B e antigen (HBeAg), international normalized ratio (INR), and prothrombin time activity (PTA), were obtained from laboratory reports. HBV DNA load was determined by PCR with a sensitivity of 500 IU/ml.

### Statistical analysis

Statistical analysis was performed using SPSS 22.0 software. Data are shown as numbers or medians and ranges. Differences between groups were analyzed using the Kruskal–Wallis test. Spearman’s tests were used for correlation analysis. Diagnostic accuracy was evaluated by the area under the receiver operating characteristic curve (AUROC). A *P* value < 0.05 was considered significant.

## Results

### The characteristics of the subjects

The general characteristics of the ACHBLF patients, CHB patients and healthy controls are described in Table 2. Patients with ACHBLF were older and showed higher degrees of AST, TBIL and INR, as well as lower levels of ALB and PTA, than healthy controls and CHB patients. Furthermore, CHB patients had higher levels of ALT and AST, than healthy controls. No significant difference in age was found between CHB patients and healthy controls. There were no significant differences in sex distribution among the groups.

**Table 2:** Baseline characteristics of the enrolled subjects

Variables	Healthy controls (*n* = 20)	CHB (*n* = 82)	ACHBLF (*n* = 31)
Age (years)	41.5 (32.25–46.75)	38.5 (32–48)	51 (44–56)^△,*^
Sex (male/female)	13/7	52/30	22/9
ALT (U/L)	28 (16–34)	161 (102–308)^△^	188 (64–962)^△^
AST (U/L)	23.5 (19.3–29.0)	104.0 (54.0-185.8)^△^	267 (83–913)^△,*^
ALB (g/L)	46.2 (37.9–51.2)	42.2 (39.5–45.0)	31.8 (28.4–34.9)^△,*^
TBIL (µmol/L)	10.9 (9.1–17.2)	15.7 (11.6–23.6)	166.0 (126.2-220.5)^△,*^
HBeAg (+/-)	NA	58/24	20/11
Log10 (HBV DNA load)	NA	7.01 (5.83–7.72)	6.51 (3.89–7.97)
PTA (%)	94.0 (84.5–105.0)	94.0 (85.0-101.3)	46.0 (35.0–52.0)^△,*^
INR	1.05 (1.00-1.12)	1.09 (1.03–1.16)	1.77 (1.57–2.15)^△,*^

CHB, chronic hepatitis B; ACHBLF, acute-on-chronic hepatitis B liver failure; ALT, alanine aminotransferase; AST, aspartate aminotransferase; ALB, albumin; TBIL, total bilirubin; HBeAg, hepatitis B e antigen; HBV, hepatitis B virus; INR, international normalized ratio; PTA, prothrombin activity; NA, not available

Data are shown as median (centile 25; centile 75) or number; *P < 0.05, compared with CHB; △*P* < 0.05, compared with healthy controls

### mRNA expression of Wnt5a, IL-6, TNF-α and IL-1β in PBMCs from ACHBLF patients, CHB patients, and healthy controls

**Fig. 2:**
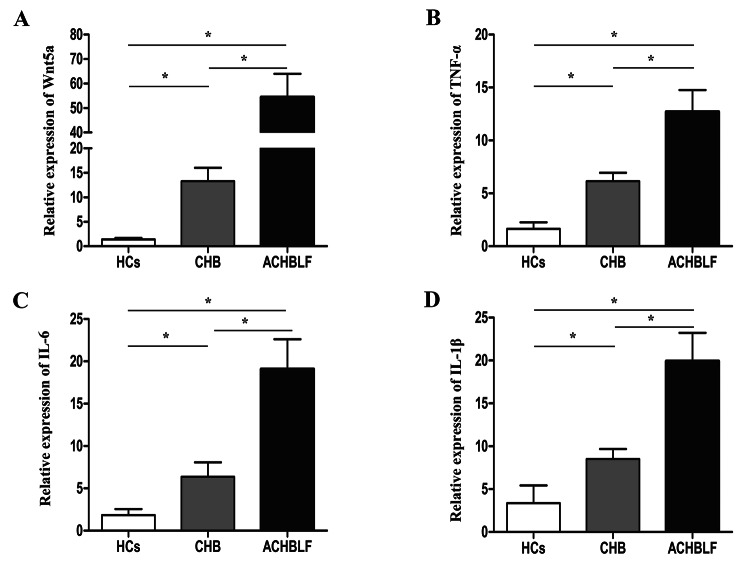
**Relative expression of Wnt5a mRNA and its associated cytokines were determined in PBMCs from ACHBLF patients, CHB patients and healthy controls.** Relative expression of Wnt5a (**A**), TNF-α (**B)**, IL-6 (**C**) and IL-1β (**D**) mRNA in PBMCs from ACHBLF patients, CHB patients and healthy controls. * *P* < 0.05

We initially analyzed the mRNA levels of Wnt5a and its associated inflammatory cytokines in PBMCs from ACHBLF patients, CHB patients and healthy controls. As shown in Fig. 2A, the level of Wnt5a mRNA was higher in CHB patients than in healthy controls (5.01 [1.63, 14.72] vs. 0.96 [0.44, 2.43], *P* < 0.01) but lower than that in ACHBLF patients (5.01 [1.63, 14.72] vs. 28.43 [15.28, 98.70], *P* < 0.01]. Similar trends were found in Wnt5a-associated inflammatory cytokine expression, including TNF-α, IL-6 and IL-1β (Fig. 2B, C and D). CHB patients had higher mRNA levels of TNF-α, IL-6 and IL-1β than healthy controls (TNF-α, 3.09 [1.69–6.62] vs. 0.73 [0.59–1.76], *P* < 0.01; IL-6, 1.70 [0.86–4.94] vs. 0.82 [0.48–1.91], *P* < 0.05; IL-1β, 4.26 [2.57-1.00] vs. 0.94 [0.59–1.77], *P* < 0.01). In particular, expression of TNF-α, IL-6 and IL-1β in ACHBLF patients was increased compared with that in CHB patients (TNF-α, 8.51 [5.13–16.10] vs. 3.09 [1.69–6.62], *P* < 0.01; IL-6, 8.98 [3.90-30.03] vs. 1.70 [0.86–4.94], *P* < 0.01; IL-1β, 17.52 [3.72–29.16] vs. 4.26 [2.57-1.00], *P* < 0.05).

### Correlation between Wnt5a mRNA levels and laboratory indices or inflammatory cytokine expression in CHB patients and ACHBLF patients

**Fig. 3:**
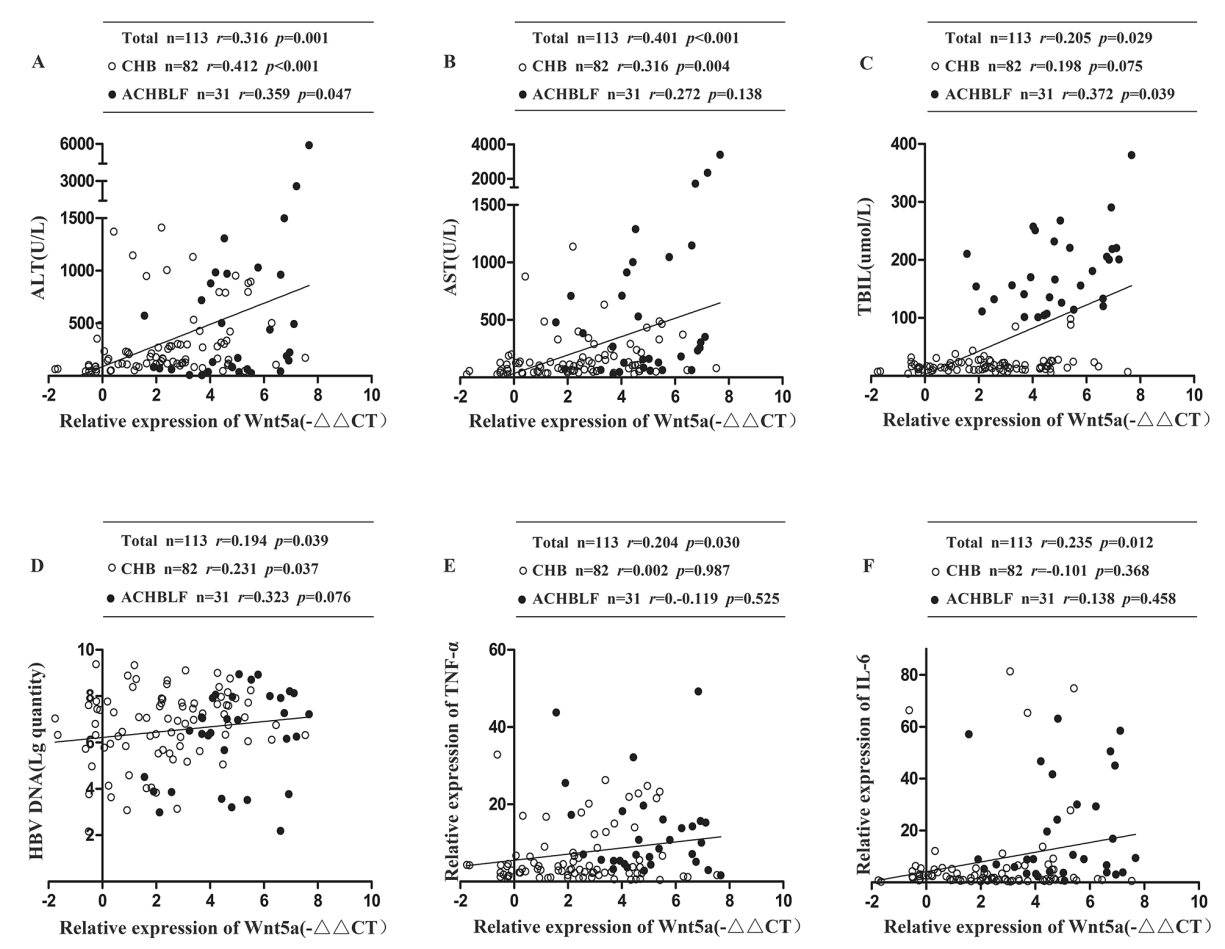
**Correlations between Wnt5a mRNA expression and liver injury markers, HBV DNA load and inflammatory cytokines were analyzed in CHB and ACHBLF patients.** Correlations between Wnt5a mRNA expression and serum ALT (**A**), AST (**B**), TBIL (**C**), HBV DNA load (**D**), TNF-α (**E**) and IL-6 (**F**). * *P* < 0.05

We applied Spearman rank correlation analysis to analyze the correlation between Wnt5a mRNA levels and laboratory indices or inflammatory cytokine expression in PBMCs from ACHBLF patients and CHB patients (Fig. 3). Our findings indicated that Wnt5a mRNA expression was positively associated with ALT (*r* = 0.316, *P* = 0.001, Fig. 3A), AST (*r* = 0.401, *P* < 0.001, Fig. 3B), TBIL (*r* = 0.205, *P* = 0.029, Fig. 3C), and HBV DNA (*r* = 0.194, *P* = 0.039, Fig. 3D) in all patients, including CHB patients and ACHBLF patients. Interestingly, we observed a positive correlation between Wnt5a and AST or HBV DNA in CHB patients (AST, *r* = 0.316, *P* = 0.004; HBV DNA, *r* = 0.231, *P* = 0.037), but there were no significant correlations in ACHBLF patients (AST, *r* = 0.272, *P* = 0.138; HBV DNA load, *r* = 0.323, *P* = 0.076; Fig. 3B and D). However, Wnt5a correlated positively with serum levels of TBIL in ACHBLF patients (*r* = 0.372, *P* = 0.039) but with no significant correlation in CHB patients (*r* = 0.198, *P* = 0.075, Fig. 3C). Furthermore, we assessed correlations between Wnt5a expression and its associated inflammatory cytokines in HBV-associated subjects. Wnt5a exhibited positive correlations with TNF-α (*r* = 0.204, *P* = 0.030; Fig. 3E) and IL-6 (*r* = 0.235, *P* = 0.012; Fig. 3F) but no significant association with IL-1β.

### Receiver operating characteristic (ROC) analysis of Wnt5a mRNA expression to discriminate ACHBLF from CHB

**Fig. 4:**
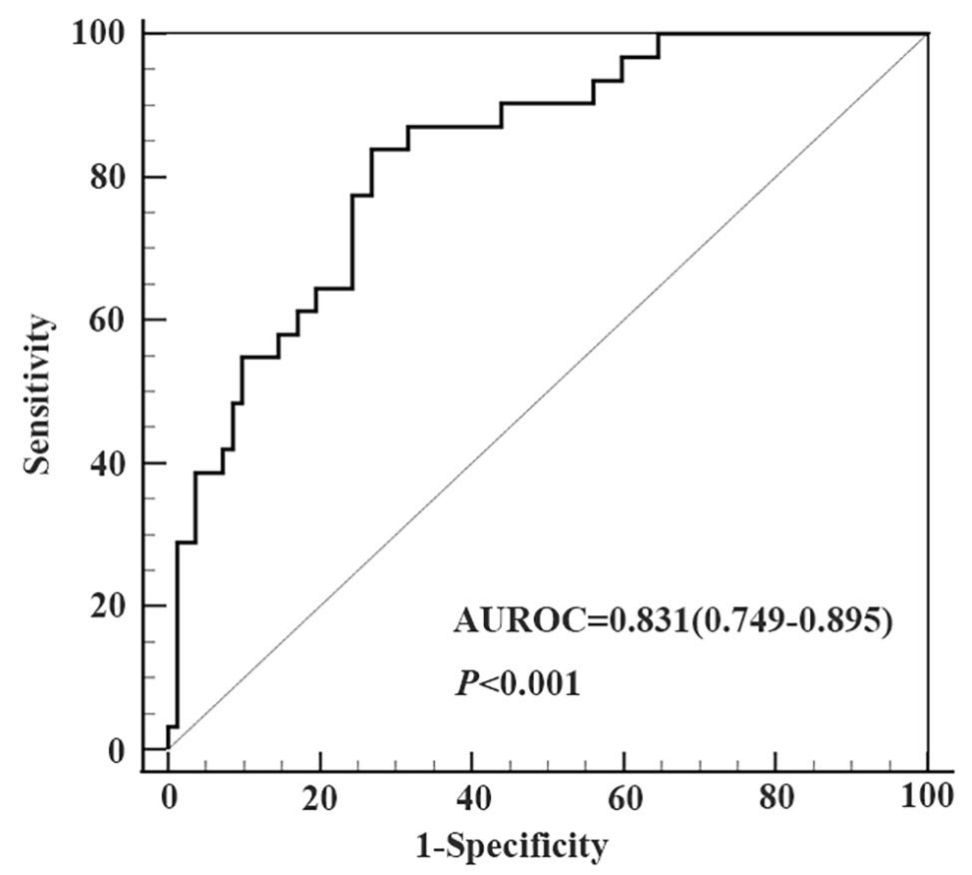
**Predicative value for Wnt5a mRNA was evaluated in discriminating ACHBLF from CHB.** The AUROC was 0.847 (95% CI: 0.734–0.925)

We performed ROC analysis to evaluate the ability of Wnt5a mRNA expression in PBMCs to distinguish ACHBLF from CHB. As depicted in Fig. 4, the area under the receiver operating characteristic (AUROC) curve of relative Wnt5a mRNA in predicting the occurrence of ACHBLF in CHB patients was 0.831 (95% CI: 0.749–0.895, *P* < 0.001). The optimal cut-off value was 12.25 for the relative Wnt5a mRNA level with a sensitivity of 83.87% and a specificity of 73.17%.

### Association between intrahepatic Wnt5a expression and inflammation in chronic HBV Infection patients

**Fig. 5:**
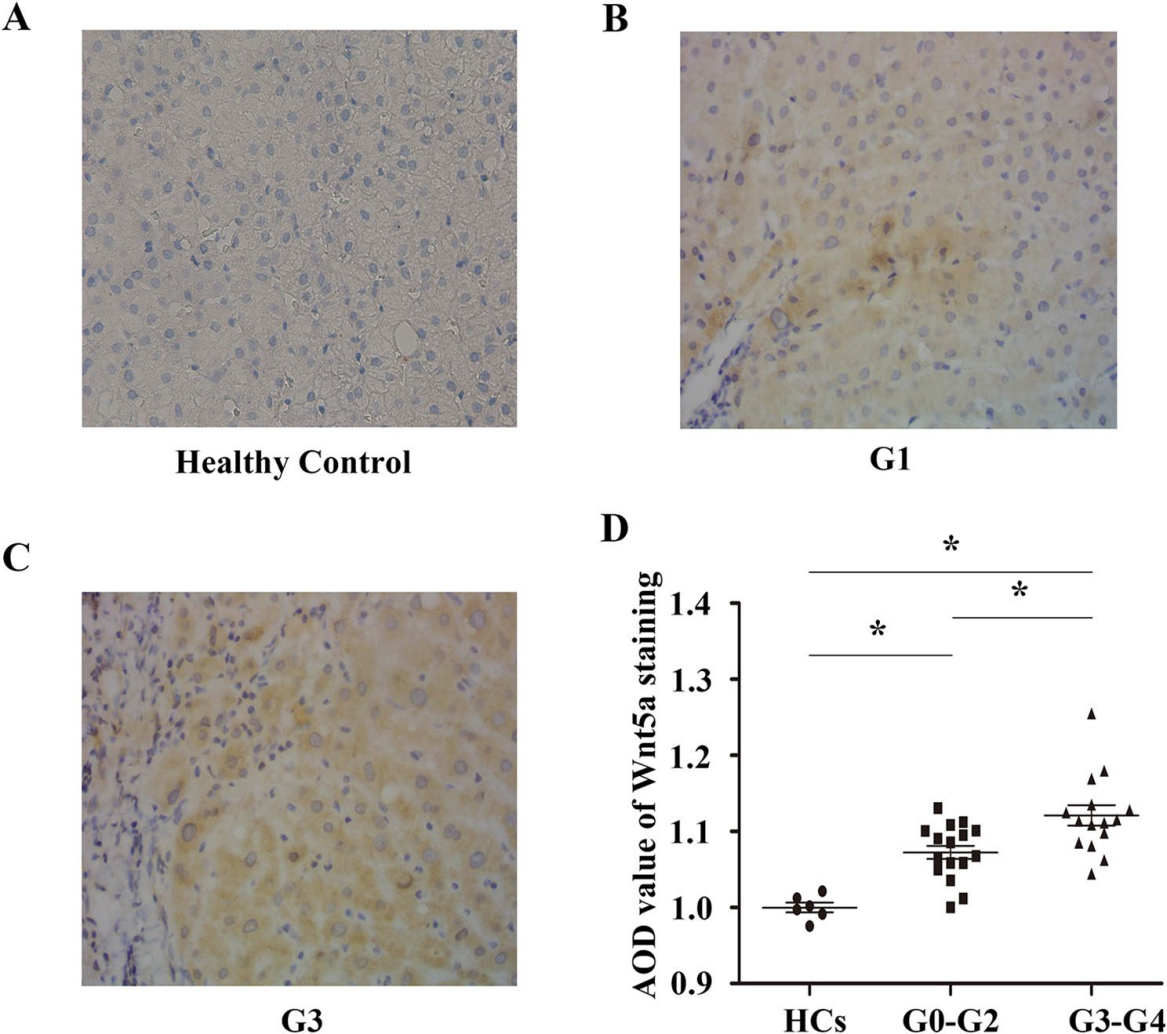
**Intrahepatic Wnt5a protein expression was determined in patients with chronic HBV infection and normal controls by immunohistochemical staining.** Representative immunohistochemical photographs for intrahepatic Wnt5a protein in normal controls (**A**) and chronic HBV infection patients with hepatic inflammatory grade scores G1 (**B**) and G3 (**C**) (magnification, × 400). **D.** Quantitative analysis of Wnt5a immunohistochemistry staining. * *P* < 0.05. AOD, average optical density

We further analyzed intrahepatic Wnt5a protein expression in 6 normal controls and 32 chronic HBV infection patients. The characteristics of these participants are shown in Tables 3 and 4. The Wnt5a protein primarily localized to the cytoplasm, as depicted in the representative immunohistochemistry images shown in Fig. 5A, B, and C. Quantitative analysis revealed that intrahepatic Wnt5a protein expression was increased in chronic HBV infection patients compared to normal controls (*P* < 0.001; Fig. 5D). Furthermore, intrahepatic Wnt5a expression in patients with higher liver inflammatory grade scores (G3-G4) was significantly elevated compared to that in patients with lower liver inflammatory grade scores (G0-G2) (*P* < 0.05; Fig. 5D). Interestingly, we found no significant difference in serum levels of ALT or AST between patients with higher liver inflammatory grade scores and those with lower scores (ALT, P > 0.05; AST, P > 0.05; Table 5).

**Table 3:** Baseline characteristics of the chronic HBV infection patients and normal controls receiving liver biopsies

variables	Chronic HBV infection patients (*n* = 32)	Normal controls (*n* = 6)	*P* value
Age (years)	39.5 (33-45.75)	37 (31.25–45.5)	> 0.05
Sex (male/female)	21/11	4/2	> 0.05
ALT (U/L)	26.0 (18.3–33.0)	21.0 (17.5–26.0)	> 0.05
AST (U/L)	21.5 (16.3–26.0)	25.0 (18.3–32.5)	> 0.05
HBeAg (+/-)	14/18	NA	
Log10 (HBV DNA load)	5.18 (2.95–7.35)	NA	
Hepatic inflammatory grade (n) G0/G1/G2/G3/G4	0/5/12/14/1	NA	

HBV, hepatitis B virus; ALT, alanine aminotransferase; AST, aspartate aminotransferase; HBeAg, hepatitis B e antigen; NA, not available. Data are shown as median (centile 25; centile 75) or number

**Table 4:** Baseline characteristics of each chronic HBV infection patient receiving liver biopsies

Case	Age	Gender	G (Inflammation)	S (Fibrosis)	ALT (U/L)	AST (U/L)
1	55	Male	1	1	33	23
2	47	Female	1	1	18	22
3	41	Male	1	1	31	18
4	47	Male	1	1	21	21
5	43	Male	1	2	13	17
6	33	Female	2	1	5	12
7	38	Male	2	1	27	23
8	53	Male	2	1	20	16
9	26	Male	2	2	20	14
10	36	Male	2	2	19	24
11	35	Male	2	2	21	16
12	34	Female	2	2	21	20
13	45	Male	2	2	32	21
14	32	Female	2	2	20	18
15	45	Female	2	2	50	26
16	49	Male	2	3	96	43
17	40	Male	2	3	32	25
18	25	Female	3	1	32	29
19	33	Male	3	2	79	35
20	58	Male	3	2	25	31
21	48	Male	3	2	16	15
22	39	Male	3	2	29	25
23	31	Female	3	2	16	16
24	46	Male	3	2	37	20
25	34	Male	3	2	78	45
26	32	Male	3	3	32	29
27	41	Male	3	3	45	23
28	34	Female	3	3	11	18
29	26	Female	3	3	33	26
30	43	Female	3	3	37	38
31	31	Male	3	4	16	10
32	45	Female	4	2	12	13

**Table 5:** Levels of serum ALT or AST in chronic HBV infection patients with different hepatic inflammatory grades

Variables	Chronic HBV infection patients with low hepatic inflammatory grade (G0-2) (*n* = 17)	Chronic HBV infection patients with high hepatic inflammatory grade (G3-4) (*n* = 15)	*P* value
ALT(U/L)	21.0(19.5–32.0)	32.0(16.0–37.0)	>0.05
AST(U/L)	21.0(16.5–23.5)	25.0(31.0–45.0)	>0.05

HBV, hepatitis B virus; ALT, alanine aminotransferase; AST, aspartate aminotransferase; Data are shown as median (centile 25; centile 75)

## Discussion

Aberrant Wnt5a expression has been observed in infection, inflammation, and immunity [17, 33, 34]. However, the role of Wnt5a in progression of chronic HBV infection has not been well investigated. The findings of the present study indicate that peripheral and intrahepatic Wnt5a expression is increased and positively associated with liver injury in patients with chronic HBV infection.

Initially, we found that Wnt5a mRNA expression was increased in PBMCs from CHB patients compared to those from HCs. In addition, intrahepatic Wnt5a protein expression was increased in patients with chronic HBV infection compared to that in normal controls. Previous studies have suggested that mutations in the X protein of HBV regulate Wnt5a expression in hepatocellular carcinoma (HCC) tissues and an HCC cell line [35]. Intrahepatic Wnt5a protein was increased in HBV-infected patients as liver inflammation and fibrosis improved, and it was upregulated by HBV replication in Hep AD38 cells [30]. Consistent with these studies, we observed that Wnt5a expression in PBMCs from CHB patients correlated positively with HBV DNA load. In our study, we also observed significantly higher mRNA levels of TNF-α, IL-6, and IL-1β in CHB patients than in HCs. A number of studies have demonstrated that Wnt5a can activate the nuclear factor-kappa B (NF-κB) and JNK pathways, leading to production of proinflammatory cytokines, including TNF-α, IL-6 and IL-1β [36, 37]. In line with these findings, we previously showed that exogenous Wnt5a stimulation dependent on JNK signaling resulted in production of TNF-α and IL-6 in THP-1 cells [29]. Furthermore, our results suggest that Wnt5a correlates positively with IL-6 and TNF-α. Therefore, our findings indicate that Wnt5a might play a potential role in the pathogenesis and progression of chronic HBV infection.

In addition to its involvement in inflammation, recent studies have highlighted the significant role of Wnt5a in tissue damage, such as lung, kidney, and myocardial injuries [36, 38, 39]. Nevertheless, the association between Wnt5a and liver injury in chronic HBV infection remains unknown. In our study, we initially observed higher levels of Wnt5a mRNA in PBMCs from ACHBLF patients than in those from CHB patients. Additionally, ACHBLF patients exhibited higher mRNA expression of IL-6, IL-1β and TNF-α than CHB patients. ACHBLF serves as an acute and severe deterioration in liver function leading to liver failure in CHB [32]. In other words, ACHBLF patients exhibited significantly more severe hepatic injury than CHB patients. Therefore, our findings suggest that an increase in Wnt5a expression is associated with deterioration of liver function in CHB patients. Furthermore, we observed that Wnt5a was positively associated with serum ALT and AST levels, which are commonly utilized as serum markers for liver injury, in CHB patients. In contrast, in ACHBLF patients, Wnt5a expression exhibited a positive correlation with TBIL levels, a crucial indicator of severe liver damage. Our findings also suggest that intrahepatic Wnt5a protein levels are increased in chronic HBV infection patients with high liver inflammatory grades (G3-G4) compared to those with low liver inflammatory grades (G0-G2) and HCs. Importantly, we made an intriguing observation that despite having normal serum ALT/AST levels, several patients with higher hepatic inflammation (G3-G4) displayed increased protein expression of hepatic Wnt5a. Consistent with our findings, previous clinical studies have suggested that CHB patients can exhibit persistently normal ALT levels despite significant inflammation in liver tissues [40, 41]. In this case, a strong correlation between Wnt5a and liver inflammation still existed. However, a larger sample is needed for validation. In summary, our results suggest that Wnt5a might be associated with liver injury during chronic HBV infection.

Moreover, we investigated the role of Wnt5a as a predictor in diagnosis of ACHBLF in CHB patients by ROC curve analysis. Our findings indicated that a cut-off value of 12.25 for relative Wnt5a mRNA expression had substantial discriminatory ability in distinguishing ACHBLF from CHB. ACHBLF is associated with considerable morbidity and mortality. Liver transplantation is the definitive and effective treatment for ACHBLF; however, its feasibility is limited by the availability of compatible donor livers [42]. Early diagnosis and timely intervention are generally managed to improve prognosis of ACHBLF [43]. Unfortunately, reliable predictors are lacking to identify early which patients with previously stable chronic HBV infection may experience progression to liver failure [44]. Our findings may offer a novel diagnostic tool for early detection of ACHBLF in CHB patients. However, the diagnostic value of Wnt5a should be validated through a larger, multicenter validation cohort.

There are some limitations in our present study. First, the sample size of subjects enrolled from our single institution was relatively small. We collected only 32 liver tissues from chronic HBV infection patients, and no liver tissues were obtained from ACHBLF patients due to their poor health condition and the limited acceptance of liver biopsy. Second, to explore the extensive role of Wnt5a in progression of chronic HBV infection, we should consider other HBV-infected diseases, including cirrhosis and HCC. A series of studies should be conducted on this issue. Recently, we investigated the prognostic role of serum Wnt5a in ACHBLF patients [28] and explored the underlying mechanism of Wnt5a contributing to acute liver failure with a mouse model [29]. Third, the precise mechanism underlying the contribution of Wnt5a to liver injury in chronic HBV infection remains obscure. Future studies should utilize Wnt5a knockout and overexpression cell lines as well as HBV transgenic mice to gain further insight.

## Conclusion

Our findings indicate a significant increase in peripheral and intrahepatic Wnt5a expression in patients with chronic HBV infection. Wnt5a expression was observed to be associated with liver injury in chronic HBV infection. Moreover, Wnt5a mRNA expression in PBMCs may serve as a predictive marker for distinguishing ACHBLF from CHB.

## Data Availability

All data generated or analyzed during the study are available from the corresponding author upon reasonable request.

## Abbreviations

HBV: Hepatitis B virus
CHB: Chronic hepatitis B
ACLF: Acute-on chronic liver failure
Fzd: Frizzled
Ror: Receptor tyrosine kinase-like orphan receptor
Ryk: Receptor tyrosine kinase related tyrosine kinase
JNK: c-Jun N-terminal Kinases
CaMKII: Calmodulin dependent protein kinase II
PKC: Protein kinase C
HAART: Highly active antiretroviral therapy
IL-6: Interleukin-6
IL-1β: Interleukin-1β
IL-8: Interleukin-1β
TNF-α: Tumor necrosis factor-α
COPD: Chronic obstructive pulmonary disease
FZD5: Frizzled class receptor 5
CCL2: C-C motif chemokine 2
PM2.5: Fine particulate matter
PBMCs: Peripheral blood mononuclear cells
ACHBLF: Acute-on-chronic hepatitis B liver failure
HCs: Healthy controls
HAI: The modified histological activity index
HBsAg: Hepatitis B surface antigen
ALT: Alanine aminotransferase
APASL: The Asian Pacific Association for The Study of the Liver
HIV: Human immunodeficiency virus
EDTA: Ethylenediaminetetraacetic
HRP: Horseradish peroxidase
DAB: Diaminobenzideine
AST: Aspartate aminotransferase
TBIL: Total bilirubin
ALB: Albumin
HBeAg: Hepatitis B e antigen
INR: International normalized ratio
PTA: Prothrombin time activity
PCR: Quantitative real-time polymerase chain reaction
AUROC: The receiver operating characteristic curve
HCC: Hepatocellular carcinoma
NF-κB: Nuclear factor-kappa B
